# Supplementary material for: Modeling the oxygen uptake, transport and consumption in an estivating terrestrial snail, Xeropicta derbentina, by the Colburn analogy
Source: PLoS One. 2021 May 20;16(5):e0251379. doi: 10.1371/journal.pone.0251379 (PMC8136638; doi:10.1371/journal.pone.0251379)
Supplement: Attachment — Submitted filename: Response to Reviewers.docx [file pone.0251379.s001.docx]

Response to Reviewers

Manuscript:

***Modeling the oxygen uptake, transport and consumption in an estivating terrestrial snail, Xeropicta derbentina, by the Colburn analogy***

by

Ulf Fischbach, Heinz-R. Köhler, David Wharam, Ulrich Gärtner

Dear Editors,

Please find our revised ms. Apart from praise, there was just a single suggestion for improvement:

*Reviewer 1: „I can accept this for the publication with a minor comment. In equation (32), the diffusion coefficient D_1 was adopted for that in the water, as the authors specified. Can it be further justified?“*

We gladly take up this suggestion and added an explanatory sentence in the revised MS (page 19, lines 2-4 in the „Track Changes“ version; page 18, last line and page 19, lines 1-2 in the „Changes Accepted“ version):

„This approximation is justified by the fact that, besides of some cells and dissolved ions, carbohydrates, lipids, glycerol, amino acids, hormones and pigments, hemolymph is basically water.“

Furthermore, we formatted our ms according tot he formatting guidelines. We hope that our revised manuscript can now be accepted for publication.

With best regards,

Sincerely,

Heinz Köhler
